# Chitosan-Coated Magnetic Nanoparticles Prepared in One Step by Reverse Microemulsion Precipitation

**DOI:** 10.3390/ijms141019636

**Published:** 2013-09-27

**Authors:** Raúl G. López, María G. Pineda, Gilberto Hurtado, Ramón Díaz de León, Salvador Fernández, Hened Saade, Darío Bueno

**Affiliations:** Centro de Investigación en Química Aplicada, Blvd. Enrique Reyna 140, Saltillo 25294, Coahuila, Mexico; E-Mails: maguapies@yahoo.com.mx (M.G.P.); gilberto.hurtado@ciqa.edu.mx (G.H.); ramon.diazdeleon@ciqa.edu.mx (R.D.L.); elcid49ster@gmail.com (S.F.); hened.saade@ciqa.edu.mx (H.S.); dario.bueno@ciqa.edu.mx (D.B.)

**Keywords:** chitosan magnetic nanoparticles, one-step microemulsion precipitation, chitosan low content

## Abstract

Chitosan-coated magnetic nanoparticles (CMNP) were obtained at 70 °C and 80 °C in a one-step method, which comprises precipitation in reverse microemulsion in the presence of low chitosan concentration in the aqueous phase. X-ray diffractometry showed that CMNP obtained at both temperatures contain a mixture of magnetite and maghemite nanoparticles with ≈4.5 nm in average diameter, determined by electron microscopy, which suggests that precipitation temperature does not affect the particle size. The chitosan coating on nanoparticles was inferred from Fourier transform infrared spectrometry measurements; furthermore, the carbon concentration in the nanoparticles allowed an estimation of chitosan content in CMNP of 6%–7%. CMNP exhibit a superparamagnetic behavior with relatively high final magnetization values (≈49–53 emu/g) at 20 kOe and room temperature, probably due to a higher magnetite content in the mixture of magnetic nanoparticles. In addition, a slight direct effect of precipitation temperature on magnetization was identified, which was ascribed to a possible higher degree of nanoparticles crystallinity as temperature at which they are obtained increases. Tested for Pb^2+^ removal from a Pb(NO_3_)_2_ aqueous solution, CMNP showed a recovery efficacy of 100%, which makes them attractive for using in heavy metals ion removal from waste water.

## 1. Introduction

Chitosan-coated magnetic nanoparticles (CMNP) contain a core of magnetic material usually a mixture of magnetite (Fe_3_O_4_) and maghemite (γ-Fe_2_O_3_). Their chitosan covering provides them with free amino and hydroxyl groups that enable the possibility to bind to a diversity of chemical groups and ions, leading to a number of applications such as protein and metal adsorption [1–3], guided drug and gene delivery [4,5], magnetic resonance imaging [6], tissue engineering [7] and enzyme immobilization [8–10]. Furthermore, this type of nanoparticle could be used in hyperthermia treatment for destroying malignant cells [11].

CMNP can be prepared by a method that involves the obtaining of the magnetic nanoparticles, usually by coprecipitation process, followed by chitosan coating [2–14]. This method allows the collection of coated nanoparticles with mean diameters ranging from 14 [14] to 25–30 nm [2,12,13] and different chitosan contents. Several research teams, included ours, have tried to simplify the procedure to obtain magnetic nanoparticles and their chitosan covering in one step [15–17]. The size range of CMNP obtained by this method is very wide. Wu *et al*. [16] reported mean diameters between 50 and 100 nm, while Hong and Rhee [15] obtained CMNP with 67 nm in mean diameter. These results contrast with the values of around 10 nm attained by our group [17]. In addition to particle size, another important feature in CMNP is the chitosan to magnetic nanoparticles weight ratio (CMR). The importance of this characteristic comes from the reduction in the saturation magnetization of CMNP under a given magnetic field induced by the covering. The higher CMR, the lower the saturation magnetization of a given amount of CMNP, recalling in this respect the high values of this ratio in the reports of Hong and Rhee [15] and Wu *et al*. [16]: 0.39 and 8.7, respectively. In these instances, only Wu *et al*. reported values of saturation magnetization at room temperature, which were less than 36 emu/g. By contrast, our group prepared CMNP with a CMR as small as 0.06 and saturation magnetization values at room temperature close to 66 emu/g [17].

Polymer coated magnetic nanoparticles with mean diameters smaller than 10 nm are of great interest because of their potential biomedical applications, such as in angiography and tumour permeability [11]. Furthermore, due to their high area to volume ratio, these ultrafine nanoparticles would be very useful in enzyme immobilization and analysis and diagnosis applications. Polymer (chitosan) coated magnetic nanoparticles with mean diameters smaller than 10 nm cannot be prepared by coprecipitation as this method is useful for obtaining larger nanoparticles. However, the method known as precipitation in reverse microemulsion allows the preparation of magnetic nanoparticles with mean diameters smaller than 10 nm [11]. It involves the preparation of a reverse microemulsion containing an aqueous phase solution of Fe salts, precursors of magnetic nanoparticles, followed by the addition of a precipitating agent [18–20]. The nanoparticles so obtained can be coated with chitosan in a further step. Precipitation in reverse microemulsions is a well-known method to prepare magnetic nanoparticles, however, as far as we know, there are no documents in the specialized literature reporting its use for obtaining CMNP in one step.

It would be advantageous to have a method that would simplify the preparation of ultrafine magnetic nanoparticles of less than 10 nm and to optimize their covering by using a thin layer of chitosan so that their magnetic properties are not diminished by the enveloping polymeric film. We report here a one-step preparation of CMNP by precipitation at 70 °C and 80 °C in a reverse microemulsion containing chitosan in the aqueous phase. These relatively high temperatures were selected due to the known direct effect of temperature on crystallinity, which in turn directly affects the nanoparticles magnetization. Based on our previous reports on preparation of CMNP with low content of chitosan in one step [17,21], appropriate amounts of this polymer were used to minimize the chitosan to magnetic nanoparticles weight ratio.

## 2. Results and Discussion

**Figure 1:**
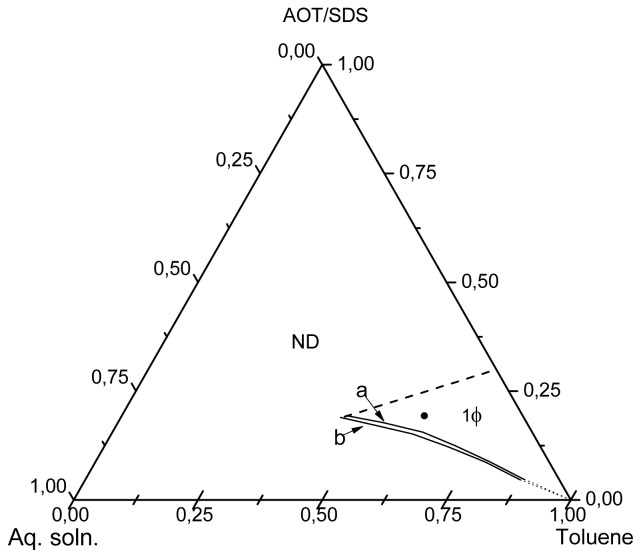
Phase diagram showing the microemulsion regions (1φ). The microemulsion composition used in the precipitation reactions is shown by (●). The limits of the microemulsion region at 70 °C and 80 °C, are represented by the lines (a) and (b), respectively. Non-determined region (ND) is not relevant for this work.

The results of the diagram phase determinations are shown in Figure 1. This diagram depicts a microemulsion region which expands from the oil rich corner toward the central part of the Gibbs’ triangle. In accordance with the position of this region, mixtures composed of lower concentrations of aqueous phase and surfactant would correspond to reverse microemulsions [22]. On the other hand, those with higher aqueous phase and surfactant concentrations would be bicontinuous microemulsions. A simple way to distinguish between a reverse and a bicontinuous microemulsions is by measuring their electrical conductivity, as the electrical current conducting capability of both differs substantially. While the conductivity of reverse microemulsions is very low, the bicontinuous microemulsions are much better electrical conductors. This difference arises from the dissimilar nanostructures dispersed in the organic phase, while reverse microemulsions contain micelles swollen with an aqueous phase, the bicontinuous microemulsions give rise to swollen interconnected channels stabilized by the surfactant. Against this background and taking into account that reverse microemulsions usually contains low aqueous phase concentrations [22], a mixture with composition inside the microemulsion region (the dot in the diagram of Figure 1), containing 15 wt% aqueous phase and 85 wt% of a mixture surfactants/toluene in a 25/75 weight ratio was prepared. The electrical conductivity measurements at 70 °C and 80 °C, gave very low values, 0.42 and 0.54 μS/cm, respectively, which are typical of a reverse microemulsion [23]. These determinations allowed the conclusion that the mixture composed of 15 wt% aqueous phase and 85 wt% of a mixture surfactants/toluene in a 25/75 weight ratio is a reverse microemulsion at 70 °C and 80 °C.

**Figure 2:**
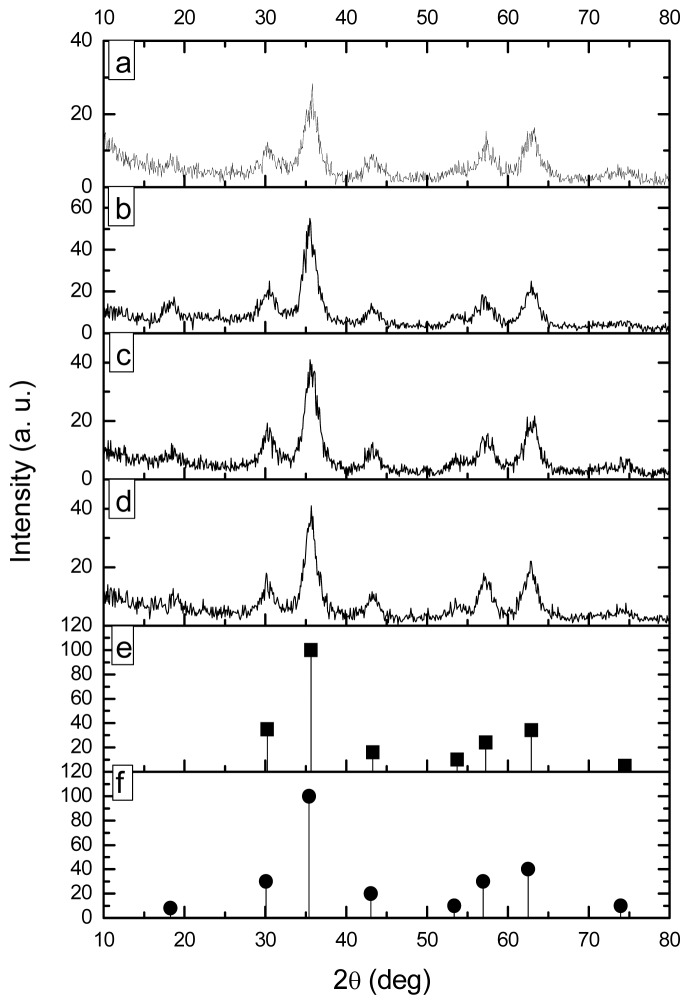
X-ray patterns of magnetic nanoparticles prepared by microemulsion precipitation: (**a**) M70; (**b**) MQ70; (**c**) M80 and (**d**) MQ80; (**e**) Magnetite and (**f**) Maghemite standard patterns are also included.

The X-ray diffraction patterns (XRDP) of the products of the precipitation reactions as well as the standard patterns of magnetite and maghemite, which were taken from the library of our X-ray equipment, are shown in Figure 2. As can be seen from this figure, both standard patterns display the same distinctive signals in the range 30° to 75° (2θ). The only difference between the patterns of magnetite and maguemite is the signal at 18.22° (2θ) showed only by the latter. This fact suggests that all the products are formed of maghemite or a mixture magnetite-maghemite. In any case, the magnetic properties of the products would be only slightly affected, taking into account that the magnetization capability of maghemite is slightly lower than that of magnetite [24].

An estimation of the average crystallite sizes of the magnetic nanoparticles obtained in the precipitation reactions can be attained by using data from their XRDP and the well-known Scherrer equation, which is represented as:

(1) $$d=\frac{K\lambda }{\beta \;\text{cos }\theta }$$

where *d* is the average diameter of the crystallite in nm; *K* is the dimensional factor (0.9); λ is the X-ray wavelength (0.154 nm); β is the line broadening at half the maximum intensity in radians, and θ is the Bragg’s angle. Table 1 shows the *d* values calculated for the reaction products. Statistically there is no difference between the sizes of the crystallite and those of the nanoparticles obtained, which indicates that the precipitation temperature and the presence of chitosan do not affect the mechanisms of crystal formation.

**Figure 3:**
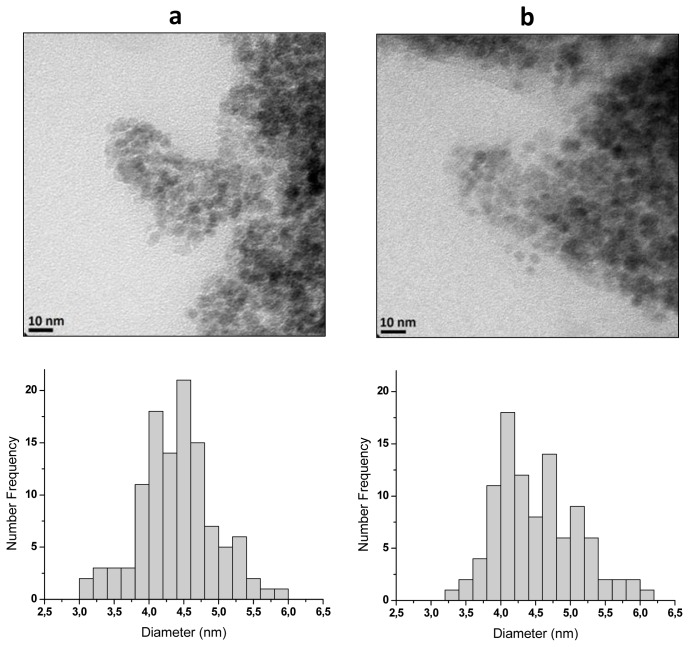
HRTEM micrographs of chitosan-coated magnetic nanoparticles: (**a**) MQ70; (**b**) MQ80. The corresponding histograms of particle sizes are also included.

While the calculations carried out by using the Scherrer equation indicate that the average crystallite diameters of the products obtained in this study are in the range 4.1 to 4.9 nm, the corresponding particle sizes are not necessarily in the same range. This is due to the possibility of crystallite aggregation, which would lead to larger nanoparticles composed of two or more crystallites. At this point in time, it is necessary to mention that a crystallite is the smallest crystal that can be formed in a given process, that is, crystals with smaller size (on average) cannot be formed. To discard (or to confirm) crystallite aggregation the results of HRTEM measurements are key. Figure 3 shows HRTEM micrographs of nanoparticles from runs MQ70 and MQ80 (Figure 3a,b, respectively) along the corresponding histograms of particle size, which were elaborated by measuring the diameter of around 120–130 particles in the set of micrographs using an image analysis program (ImageJ). From these data, *D_w_*, *D_n_* and *PDI* (*D_w_*/*D_n_*), being *D_w_* and *D_n_* the weight- and number-average diameters and *PDI* the polydispersity index, which were calculated using the following equations:

(2) $$D_{n}=\frac{\sum_{i}n_{i}D_{i}}{\sum_{i}n_{i}}=\frac{\sum n_{i}D_{i}}{n}$$

(3) $$D_{w}=\frac{\sum_{i}n_{i}D_{i}^{4}}{\sum_{i}n_{i}D_{i}^{3}}$$

where *n_i_* is the number of particles of size *D_i_* and *n* is the total number of measured particles.

*D_n_* and *PDI* values obtained for the nanoparticles from MQ70 and MQ80 precipitation reactions are included in Table 1. As can be seen there is practically no difference between the corresponding *D_n_* and *d_av_* values, which suggests the absence of crystallite aggregation and that on average each magnetic nanoparticle is composed of one crystallite. Furthermore, *D_n_* values for MQ70 and MQ80 are practically the same (≈4.5 nm), which discards the effect of temperature on particle size of magnetic nanoparticles. Worthy of note is the very small particle size that this method renders. In fact, while nanoparticles with this order of size was expected, due to the well-known capability to produce magnetic nanoparticles with average diameters smaller than 10 nm of the precipitation in reverse microemulsions method [11,18–20], there was some doubt about the modification of the mechanism of particle growth in the presence of chitosan. However, as can be seen, this variable did not affect the final size of the particles. The elucidation of the chitosan fate at the end of the process is shown in the following paragraphs.

**Figure 4:**
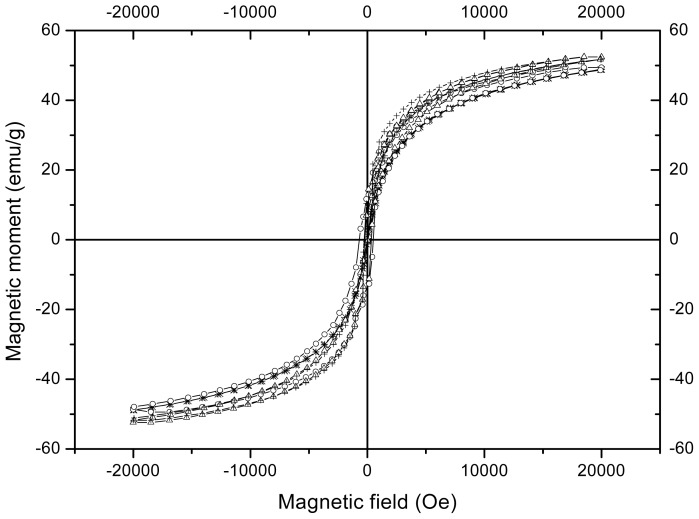
Magnetization curves at room temperature of magnetic nanoparticles prepared by microemulsion precipitation: (*) M70; (○) MQ70; (Δ) M80; (+) MQ80.

Figure 4 shows the magnetization curves at room temperature of the nanoparticles obtained with and without chitosan. The response variables characterizing the magnetic behavior of the nanoparticles are shown in Table 2. It is noteworthy that the curves in Figure 4 do not attain magnetic saturation. This phenomenon is explained as a consequence of the difficulty for aligning the magnetic moments in the direction of the applied magnetic field experimented by the surface atoms, which constitute a significant fraction of the total atoms in very small particles [25]. The data in Table 2 indicate that the nanoparticles attained magnetizations around 50 emu/g at 20 kOe and there is no statistical difference between the values of naked and those of the nanoparticles obtained in the presence of chitosan, which suggests a low chitosan content in the latter. However, the statistical similarity in magnetization values in the case of nanoparticles at 80 °C and almost for those prepared at 70 °C can be ascribed to at least two causes. One of them would be that the chitosan layer on the nanoparticles is so small that to statistically detect the magnetization decrease a large number of replicates would be required. The other cause is that an increase in the magnetite to maghemite ratio could be promoted by the presence of chitosan during the precipitation reaction. Nevertheless, a slight direct effect of temperature on magnetization can be identified. Taking into account the absence of effect of temperature on the crystallite size, the higher magnetization observed in the nanoparticles prepared at 80 °C could be related to a higher degree of crystallinity [26]. Another highlighting aspect of the magnetic properties of the nanoparticles obtained in this study is the superparamagnetism that they exhibit as concluded from the very low values of remnant magnetization and coercivity shown in Table 2. This is a typical property of magnetite and maghemite nanoparticles with diameters smaller than 10–15 nm [27]. One of the most attractive features of the superparamagnetic nanoparticles is the demagnetization of the material once the applied magnetic field is removed, which allows their reuse.

On the basis of the above results, the nanoparticles obtained in the presence of chitosan in this study show magnetization values lower than those of nanoparticles prepared also by our group by a one-step method comprising coprecipitation in the presence of low chitosan content (*ca.* 65–66 emu/g) [17,21]. Considering that the average nanoparticle diameters obtained in our previous studies were around 9.5–11 nm and that similar chitosan concentrations in the aqueous phase were used in both types of precipitation reactions (≈0.1–0.125 wt%), only the lower size of the nanoparticles from microemulsion precipitation can account for their lower magnetization values. This assumption is confirmed by the well-known direct dependence between magnetization of small particles (diameters smaller than *ca.* 15 nm) and particle size [28,29].

**Figure 5:**
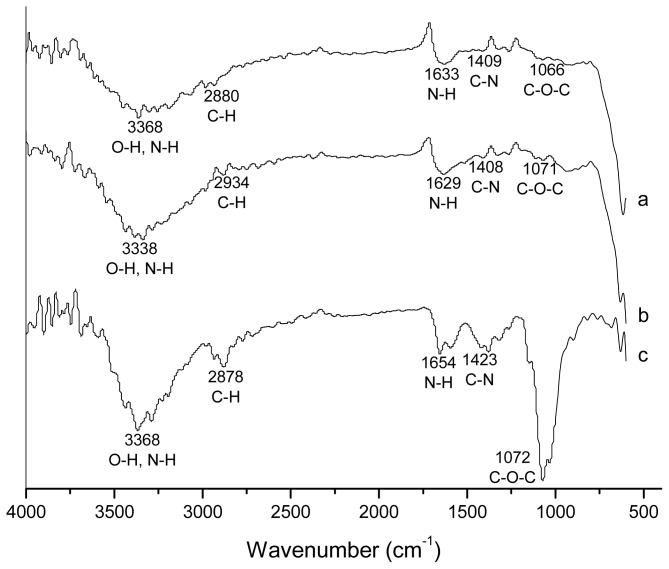
FTIR transmission spectra of magnetic nanoparticles prepared by microemulsion precipitation: (**a**) MQ70 and (**b**) MQ80; (**c**) Chitosan spectra is also included.

Figure 5 shows the FTIR spectra of pure chitosan and magnetic nanoparticles prepared with chitosan (MQ70 and MQ80). The spectrum of the products from runs MQ70-R and MQ80-R (not shown) displays a similar signal pattern to that from original runs. In accordance with that reported in the literature [30,31], the characteristic absorption bands for chitosan in Figure 5 appear at 3368 (O–H and N–H stretching vibrations), 2878 (C–H stretching vibrations), 1654 (N–H bending vibrations), 1423 (C–N stretching vibrations) and a group of bands from 1100 to 1020 cm^−1^ (C–O–C and C–O stretching vibrations). The spectra of the products obtained using chitosan show the characteristic absorption bands for this polymer, with a noticeable decrease of the group of bands due to C–O–C and C–O centered around 1050 cm^−1^. This is assigned as a C–O stretching band in the polymer [32,33] and particularly to the C–O stretch band of the secondary alcohol groups; the group of Ramalho Mercê [34] suggests that the intensity reduction of the band in similar systems may be due to coordination of the hydroxyl neighbor of the amine group in position 3. In our case, due to the samples containing a very low proportion of chitosan, our observation is in agreement with the reported results, possibly indicating that most of the chitosan is forming complexes and that small amounts of uncomplexed chitosan remain in the obtained nanoparticles. Considering that the products of the precipitation reactions in presence of chitosan were exhaustively washed and magnetically recovered, free chitosan (if there was some remaining), would remain dispersed in the discarded aqueous phase and only that attached to the magnetic nanoparticles would appear in the final product. Thus, it was concluded that all the chitosan in the final product is chemically bound to the surface of magnetic nanoparticles. However, the possibility that some magnetic nanoparticles remained uncoated cannot be dismissed.

Once the FTIR results demonstrated that the precipitation reactions carried out with chitosan rendered magnetic nanoparticles coated by this polymer, the following step was to quantify the fraction of chitosan, surfactant, if any, and magnetic material on the coated magnetic nanoparticles. In this task, carbon and sulfur contents determined by the combustion method were used. The purpose of these measurements was to estimate the chitosan and surfactant contents in the final products from the carbon and sulfur contents, respectively. For MQ70 and MQ70-R samples 2.63% and 3.01% in carbon content respectively, were obtained. On the other hand, MQ80 and MQ80-R showed values of 3.03% and 3.24%, respectively. Regarding sulfur content, values smaller than 0.02% were obtained in all cases. From the carbon content in the products and a calculated value of 45.23% for carbon content in a repetitive unit of chitosan with 75% deacetylation degree, chitosan contents of 5.8%, 6.7%, 6.7% and 7.2% for MQ70, MQ70-R, MQ80 and MQ80-R, respectively, were obtained. Because of the very low sulfur content in the products, the surfactant content in the coated magnetic nanoparticles was taken as negligible, thus concluding that the coated magnetic nanoparticles were composed only of chitosan and magnetic material. From the results above, the amount of immobilized chitosan on nanoparticles was calculated. For the analyzed products these values ranged from 61.6 to 77.6 mg chitosan/g magnetic material, which are higher than the theoretical value (≈53.0 mg/g) assuming 100% conversion to magnetite in the precipitation reaction and all chitosan in the recipe covering the nanoparticles. The higher than theoretical values of immobilized chitosan probably arise from a precipitation reaction conversion slightly lower than 100% and the formation of a mixture of magnetite and maghemite instead of only magnetite.

Up to this point, it has been proven that the precipitation reactions carried out in reverse microemulsions in the presence of chitosan render chitosan-coated magnetic nanoparticles. Nucleation and growth of magnetic particles in precipitation reactions carried out in reverse microemulsions occur through a well-known mechanism [35]. In accordance with the results given above, this mechanism is not perturbed by the presence of chitosan. It is believed that the crystals of magnetic nanoparticles are formed very quickly and that chitosan links them through the chemical bonds NH_2_–Fe, NH_2_–O and OH–Fe in a further step as a result of collisions between micelles containing nanoparticles and those containing chitosan. However, the chitosan diffusion from the latter to the former may not be discounted in the process that brings together both species.

The quantification of the amino groups on the surface of CMNP and the assessment of their reacting availability is needed to ascertain the practical application of the material. To this purpose, an estimation of the number of amino groups per each coated magnetic nanoparticle was carried out, through the following sequence of calculation. From *D_n_* value of the coated magnetic nanoparticles determined by HRTEM the magnetic material and chitosan contents and the density values of 5.2 g/mL for magnetite [36] and 1.34 g/mL for chitosan [37], the number of nanoparticles per one gram of CMNP was calculated. With this result and that of the amino groups concentration in one gram of CMNP obtained by the ninhydrin method, the number of amino groups per each coated magnetic nanoparticle was obtained. This calculation sequence was used with the data from all the precipitation reactions carried out in the presence of chitosan. As an approximation, the *D_n_* values of MQ70-R and MQ80-R nanoparticles, were considered equal to those of the MQ70 and MQ80 nanoparticles. Table 3 shows the estimated values of amino groups per each coated magnetic nanoparticle along the chitosan layer thickness, the number of nanoparticles per gram of CMNP and the amino groups concentration in one gram of CMNP obtained by the ninhydrin method. As can be seen, the chitosan layer on the magnetic nanoparticles is very small (<0.2 nm), which would almost correspond to a molecular monolayer, in the case of a homogeneous distribution of the polymer on the surface of the nanoparticles. However, the highlighting result is the number of amino groups per each coated magnetic nanoparticle: 11–12 for nanoparticles prepared at 70 °C and around 14 for those obtained at 80 °C. These values are much lower than the value of 135, corresponding to the chitosan-coated magnetic nanoparticles previously obtained by our group when the one-step method comprising coprecipitation was used [21]. In the first instance, this could be assigned to the smaller particle size that precipitation in reverse microemulsions produces. However, by comparing the mean sizes of the particles from both methods, the reduction factor was only close to 2.2, which leads to an area per particle reduction factor of 4.84. Considering an equal number of amino groups per area unit, the nanoparticles obtained by microemulsion precipitation should contain nearly 28 amino groups per particle. The lower values obtained suggest that chitosan is not homogeneously distributed on the nanoparticles forming an almost monomolecular layer, hinting the formation of aggregates in some regions on the nanoparticle surface, which in turn reduces the chitosan surface and the number of the exposed amino groups. In fact simple calculations show that on average each nm^2^ of surface of MQ70 and MQ80 nanoparticles contains around 0.2 exposed amino groups. By comparison the CMNP obtained by the one-step method comprising coprecipitation had a value of 0.42 exposed amino groups.

To evaluate the reacting availability of the amino groups on the surface of CMNP, runs for Pb^2+^ removal from a Pb(NO_3_)_2_ aqueous solution by the nanoparticles from MQ70 and MQ80 precipitation reactions were carried out. This test is based on the ability of chitosan to chelate heavy metal ions through its amino groups. The results of the runs carried out with both type of nanoparticles showed that 96.8% of the Pb^2+^ ions were chelated by the MQ70 sample after only 10 min and at 40 min, the concentration of Pb^2+^ ions in the solution was under the detectable level of the apparatus (0.025 ppm), which was equivalent to chelating efficiency >99.8%. On the other hand, the MQ80 sample showed a better behavior due to the fact that the Pb^2+^ ions were undetectable after only 10 min of treatment. Calculations determined that the number of all Pb^2+^ ions contained in the initial solution was 1.45 × 10^18^. On the other hand, from data in Table 3 and the CMNP amounts used in the Pb^2+^ removal runs, it was calculated that the amino groups on the surface of all dispersed nanoparticles were 1.38 × 10^18^ and 1.66 × 10^18^ for MQ70 and MQ80 nanoparticles, respectively. From these quantities a ratio of amino groups to chelated Pb^2+^ ions was found to be around one for both MQ70 and MQ80 nanoparticles, which roughly means that each amino group on the surface of the nanoparticles chelated one Pb^2+^ ion. Compared to the results previously obtained by our group with CMNP prepared by a one-step method comprising coprecipitation [21] this finding is highly surprising. In our prior work we found the ratio of amino groups to chelated Pb^2+^ ion to be 3.4. Furthermore, only around 70% of initial Pb^2+^ ions were chelated at the end of the run (50 min). A possible explanation for the greater efficiency in Pb^2+^ capturing showed by the CMNP prepared by microemulsion precipitation could arise from the applied sonication during the runs. It is probable that the ultrasonic energy applied in our previous study was not enough to deagglomerate the CMNP clusters in the aqueous dispersion, which led to a reduction in the total surface area and as a consequence to a reduction of the available amino groups capable of chelating the Pb^2+^ ions.

Although indirectly these results would demonstrate the re-dispersion feasibility of the dried CMNP in an aqueous solution, newly prepared CMNP samples, synthesized at 70 °C and 80 °C, were brought to dryness and re-suspended under similar conditions to those used in the Pb^2+^ removal test. The QLS measurements of samples of these dispersions gave mean particle diameters ranging 200–250 nm, indicating the dried CMNP propensity to aggregate forming clusters. However, it is believed that Pb^2+^ ions would diffuse into the interstices of the clusters to reach the amino groups on the nanoparticles surface thus maintaining their complexing properties.

## 3. Experimental Section

### 3.1. Materials

Chitosan with low molecular weight and 75% deacetylation degree, ferric chloride (FeCl_3_·6H_2_O, 99%), ferrous chloride (FeCl_2_·4H_2_O, 98%), aqueous ammonia (NH_4_OH, 57.6 wt%), sodium dodecyl sulfate (SDS, 98%), sodium bis (2-ethylhexyl) sulfosuccinate (AOT, 98%) and ninhydrine (97%) from Aldrich were used as received. Lead nitrate, Pb(NO_3_)_2_, 99.7%, from J.T. Baker, was also used as received. De-ionized and triple-distilled water was drawn from a Millipore system.

### 3.2. Phase Diagram Determinations

Microemulsion regions at 70 °C and 80 °C were determined by titration with 0.25 M aqueous solution of a FeCl_3_·6H_2_O/FeCl_2_·4H_2_O mixture (3/2, mol/mol) of solutions of the surfactant (AOT/SDS, 2/1, *w*/*w*)/toluene at the following weight ratios: 5/95, 10/90, 15/85, 20/80 and 25/75. Phase boundary was detected visually at each one of the constant (AOT/SDS)/toluene lines studied. Then, samples with compositions slightly below and above that of the visually determined phase boundary were prepared by weighting each component and allowing to reach equilibrium in a water bath at 70 °C and 80 °C to determine more precisely the phase boundary. The one phase microemulsion region corresponds to transparent or translucent samples that do not exhibit birefringence when observed through cross polarizers.

### 3.3. Preparation of Magnetic Nanoparticles

The different types of precipitation reactions, each one in duplicate, were carried out in a 150 mL jacketed glass reactor equipped with a reflux condenser an inlet for aqueous ammonia feed and a mechanical agitator operated at 300 rpm. To carry out the reactions, 100 g of the corresponding microemulsions were formed by mixing at the predetermined precipitation reaction (70 °C or 80 °C) 21.25 wt% (AOT/SDS, 2/1, *w*/*w*), 63.75 wt% toluene and 15.00 wt% 0.25 M aqueous solution of a FeCl_3_·6H_2_O/FeCl_2_·4H_2_O mixture (3/2, mol/mol) containing 0.1 wt% chitosan. For comparison, precipitation reactions without chitosan were also carried out. After microemulsion formation, a shot of 2.3 g aqueous ammonia was added to the reactor allowing the reaction proceed for 30 min. At the end of the reaction, the particles were recovered by using a permanent magnet, washed at least 10 times with water-acetone (81/19, *w*/*w*) and then dried. Table 4 shows the distinctive features of each one of the precipitations reactions.

### 3.4. Characterization

Electrical conductivity was measured at 70 °C and 80 °C and 1 KHz with a Hach sension 5 conductivity meter. X-ray analyses of the products were carried out with a Siemens D-5000 diffractometer using Cu-K_α_ (λ = 1.5418 Å) as incident radiation. The size and morphology of the particles were determined in a high-resolution transmission electron microscope (HRTEM) Titan-300 kV for which samples were prepared by dispersing the resulting powders in water with ultrasonication and then depositing the dispersion on a copper grid. The carbon and sulfur content in the nanoparticles were determined by the combustion method carried out in a induction furnace Eltra CS800. The magnetic properties of the nanoparticles were determined using a Physical Properties Measurement System from Quantum Design, model 6000 in mode vibrating sample magnetometer (VSM), with an applied field between −20.0 to 20.0 kOe at room temperature. Fourier transform infrared spectrometry (FTIR) was carried out in a Magna IR 550 from Nicolet with germanium crystal. The amino groups on the surface of CMNP were determined by the ninhydrin method, using glycine to construct the calibration curve [38]. To make the measurements, 0.1 g of CMNP were dispersed in 1 mL of water and then 0.6 mL of ninhydrin reagent was added. After that, the dispersion temperature was boiled for 30 min. The amino groups concentration in the dispersion was determined by readings of absorbance at 570 nm.

### 3.5. Pb^2+^ Removal Test

Typically, 24 mg of dried CMNP were added to 50 mL of a Pb(NO_3_)_2_ aqueous solution containing 10 ppm of Pb^2+^. Then, this mixture was ultrasonicated for 50 min at room temperature taking samples during the process each 10 min. After CMNP were removed, the concentration of Pb^2+^ in the samples was measured by atomic absorption spectroscopy in Varian SpectrAA 220 equipment.

## 4. Conclusions

It was proven that is possible to obtain chitosan-coated superparamagnetic nanoparticles in one step by modifying the precipitation in reverse microemulsions method. The dissolved chitosan in the aqueous phase of the microemulsion does not perturb the mechanism of nucleation and growth of magnetic nanoparticles. It is believed that the chitosan covering is a process that occurs once the magnetic nanoparticles have been formed. The coated magnetic nanoparticles obtained in this study are very small, with near 4.5 nm in mean diameter, including a very thin chitosan layer. As a consequence of this feature the final magnetization of the coated nanoparticles is practically equal to that of naked nanoparticles. It is noteworthy that despite the nanoparticles smallness the attained magnetization values are relatively high (≈50 emu/g), probably due to a high magnetite content in the mixture magnetite-maghemite in nanoparticles. Additionally, the chitosan-coated magnetic nanoparticle obtained in this study showed high efficacy in recovering Pb^2+^ ions in aqueous solution, which opens the possibility for using them in applications involving the recovery of heavy metals.

## Figures and Tables

**Table 1:** Mean sizes of grain and particle from X-ray and HRTEM measurements, respectively.

Size characteristics	M70	M70-R	M80	M80-R	MQ70	MQ70-R	MQ80	MQ80-R
*D*	4.17	4.69	4.37	4.63	4.29	3.96	4.93	4.93
*d_av_*	4.43 ± 0.37		4.40 ± 0.33		4.13 ± 0.23		4.93	
*D_n_*	-	-	-	-	4.42	-	4.51	-
*D_w_*	-	-	-	-	4.61	-	4.80	-
*PDI*	-	-	-	-	1.04	-	1.06	-

**Table 2:** Results from magnetic measurements of nanoparticles.

Precipitation temperature (°C)	Without chitosan			With chitosan		

	Magnetization (emu/g)	Remnant magnetization (emu/g)	Coercivity (Oe)	Magnetization (emu/g)	Remnant magnetization (emu/g)	Coercivity (Oe)
70	48.9 ± 0.1	0.1 ± 0.1	7.9 ± 6.0	50.1 ± 1.0	0.1 ± 0.0	7.8 ± 2.8
80	52.7 ± 0.4	0.2 ± 0.0	11.7 ± 1.1	52.9 ± 1.6	0.1 ± 0.0	6.1 ± 1.3

**Table 3:** Surface features and nanoparticle number per mass unit of the chitosan-coated magnetic nanoparticles.

Run	Chitosan layer thickness (nm)	Nanoparticles per gram of CMNP × 10^−18^	NH_2_ (mmoles) per gram of CMNP 1	NH_2_ (groups) per particle
MQ70	0.15	5.0	0.0931	11.3
MQ70-R	0.17	5.1	0.0994	11.8
MQ80	0.18	4.8	0.1118	14.2
MQ80-R	0.18	4.8	0.1102	13.9

1 determined by the ninhydrin method.

**Table 4:** Distinctive features of the precipitation reactions.

Reaction parameters	M70	M70-R	M80	M80-R	MQ70	MQ70-R	MQ80	MQ80-R
Precipitation temperature (°C)	70	70	80	80	70	70	80	80
Chitosan	No	No	No	No	Yes	Yes	Yes	Yes

The suffix R is used to identify the replicates.
